# High-level military and sport leaders’ everyday challenges and psychological skills: A cross-contextual repeated measures study

**DOI:** 10.1080/08995605.2024.2376970

**Published:** 2024-07-31

**Authors:** Andreas Bencker, Gerry Larsson, Maria Fors Brandebo, Urban Johnson, Andreas Ivarsson

**Affiliations:** a School of Health and Welfare, Halmstad University, Halmstad, Sweden; b Department of Leadership, and Demand & Control, Swedish Defence University, Stockholm, Sweden; c Department of Health and Welfare, Inland University College of Applied Sciences, Elverum, Norway; d Department of Sport Science and Physical Education, University of Agder, Kristiansand, Norway

**Keywords:** High-level military and sport leaders, demanding conditions, leader performance, mental health, psychological skills

## Abstract

Research shows that high-level military and sport leaders share a high-stress and high-stakes leader role due to similar experiences of demanding conditions mainly manifested in psychological burden. This raises research questions about leaders’ psychological strategies to maintain their mental health and performance under demanding conditions. Thus, the current study investigated how experienced demanding conditions were related to self-rated leader performance level and mental health indicators among high-level military and sport leaders and whether the application of psychological skills by these leaders moderated these relationships. A composite questionnaire was used to collect data longitudinally, once a week for four consecutive weeks. Fifty-two Swedish high-ranking military officers and executives in elite team sport organizations completed the questionnaire. Multilevel analysis revealed no effect of demanding conditions on leader performance, but they harmed leader vitality and were associated with higher stress symptoms. Moreover, psychological skills did not moderate the relationship between demanding conditions and leader performance. However, motivational and instructional self-talk negatively moderated the relationship between demanding conditions and vitality. In contrast, emotional regulation, comprised of mindfulness and cognitive reappraisal, positively moderated vitality. Emphasizing the nuanced application of psychological skills is crucial while avoiding one-sided beliefs about their positive effects. Interventions are suggested to focus on vitality and related psychological skills to ensure leaders feel good while performing under demanding conditions. More cross-contextual leadership research, suggestively applied research, is needed to better understand the links between high-level military and sport leaders’ psychological skills, leader performance, and mental health under demanding conditions.

**What is the public significance of the article?—**This cross-contextual repeated measures study contributes novel insights into high-level military and sport leaders’ who continue to perform regardless of demanding leadership challenges and impaired mental health. Moreover, they utilize mindfulness and cognitive reappraisal as a unified psychological skill to regulate their emotions, which improves vitality. Surprisingly, an increase in instructive and motivating self-talk negatively affects their mental health. Intervention among high-level leaders is suggested to adopt an enhanced focus on vitality and related psychological skills to make leaders, in addition to performing well, feel better under demanding conditions.

## Introduction

In recent years, the research literature has recognized common and collaborative research interests between the military and sport contexts. Examples of collaborative areas found in the literature related to performance psychology (Goodwin, 2008) are the value and improvement of psychological skills (DeWiggins et al., 2010), and the maintenance of mental health to feel good and perform well (Wagstaff & Leach, 2015). Moreover, leadership issues related to demanding conditions (Bencker et al., 2022) are also examples of collaborative research areas between the military and sports. With a specific focus on simultaneously exploring similarities between high-ranking Swedish military officers and sport executives, Bencker et al. (2022) revealed that the leaders shared a high-stress and high-stakes leader role due to similar experiences of their most demanding conditions, for example, “developing organizations: leading under extensive workload, and responsibility, and periods of extreme concentration: leading critical coordination, decisions, and timing” (p. 31). Although these most demanding conditions were experienced as exciting and challenging, they were primarily manifested by “troublesome cognitions, emotional discomfort, and bodily tension” (Bencker et al., 2022, p. 34). These manifestations raise research questions about high-level military and sport leaders’ psychological strategies to maintain their mental health and leader performance under demanding conditions. As previous research shows that high-level military and sport leaders share similar experiences in demanding conditions, this may also lead to shared health and leader performance challenges for them, as well as the use of effective psychological strategies to manage these challenges.

For example, research displays that elevated stress arising from work-related interpersonal relationships, multiple roles, and responsibilities is associated with challenges with self-perceived impaired leader performance, such as worsened emotional outbursts among sports coaches (Frey, 2007), indicating leaders’ challenges in managing their stress, anxiety, and accomplishment. Comparable results regarding performance deterioration under demanding conditions are found among military leaders (Larsson, 1989; Westman & Eden, 1996). For such reasons, anxiety management techniques and psychological skills adapted from sport psychology have been considered and applied in the military context. According to Hourani et al. (2011), military personnel appear to value the training of these techniques and skills as a preventive measure, which has the potential to reduce the negative impact of combat stress and serves as preparation for upcoming leadership challenges. Consequently, it is essential to examine how different psychological stress and performance management strategies affect leaders’ promotion of their mental health and leadership under demanding conditions.

To examine the relationship between demanding conditions, leader performance, and mental health and focus on psychological skills as moderators, Adler and Castro’s (2013) occupational health model for military mental health serves as a framework in the current study. The model emphasizes the importance of understanding the demanding conditions that a person is exposed to. Adler and Castro (2013) list three types of demanding conditions. Personal outside work, traditional occupational, and high-risk/traumatic work situations are involved. Adler and Castro’s model underlines the leader’s active role, using their personal resources, like psychological skills, to manage the impact of demanding conditions with its inherent leadership challenges on the leader’s mental health and performance. Another factor in the model is the leader’s background, for example, experience and rank, where high rank is claimed to be a basis for better mental health. The model also shows that personal resources in joint action with occupational resources, for example, training and intervention, can ultimately influence the effects of demanding conditions on mental health and performance.

As stated earlier, demanding conditions can negatively impact leaders’ performance and mental health. There also seems to be a covariation between mental health and leader performance. Evidence shows that leaders who have more severe degrees of emotional, cognitive, and physiological stress symptoms, indicating depression and anxiety, exhibit a higher level of destructive leadership (Byrne et al., 2014). Correspondingly, leaders’ own work stress is associated with their destructive leadership (Tafvelin et al., 2022), and their destructive leadership is negatively associated with their own well-being (Kaluza et al., 2020). In line with this leadership rupture, leader strains threaten the consistent performance of functional leadership behaviors (Klebe et al., 2022). Moreover, leaders in sports face a multitude of pressures, many of which tend to harm their mental health (Potts et al., 2023). Overall, increased stress appears to impair leaders’ mental health and leader performance. However, this evidence is not clear-cut.

Although leaders may exhibit psychological distress, they may also exhibit positive levels of mental health under demanding conditions. For example, in a study by Baldock et al. (2022), sports leaders indicated positive adaptations to some perceived challenges, as well as an increase in psychological well-being. Moreover, military leaders holding high-level positions have fewer stress reactions due to experiencing more control than non-leaders (Sherman et al., 2012). They can also be hardy because of their extensive military experience (Kokun et al., 2023). Moreover, higher self-rated overall health is shown to be linked with being in a military leadership role when deployed (Mulligan et al., 2010). Similarly, Wallis et al. (2021) found that even if high-level leaders (leaders from healthcare, public service, etc.) reported more challenges at work, they self-reported better mental health in terms of flourishing and life satisfaction, and lower psychological distress than coworkers.

Like flourishing, vitality is a positive mental health state since individuals who express vitality under demanding conditions often experience reduced psychological distress (Tremblay et al., 2006). However, it has only been vaguely investigated how vitality is affected by demanding conditions. Nevertheless, based on the proximity between vitality and well-being (Ryan & Frederick, 1997), it makes sense to highlight related research (mainly cross-sectional studies), on professional first responders (Larsson et al., 2016), military veterans (Larsson et al., 2021), and sport coaches (Richman, 1992), which shows that their psychological well-being decreases when work-related daily hassles rise. Furthermore, it is crucial to emphasize leaders’ well-being, as research shows a strong correlation between leaders’ positive well-being and both their fruitful leadership (Kaluza et al., 2020) and performance of transformational leadership behaviors (Geibel, 2022).

The following will summarize research on the psychological skills of self-talk, imagery, goalsetting, and emotional regulation through mindfulness and cognitive reappraisal and their effects on performance and mental health. Regarding self-talk, research shows that constructive self-talk reduces work pressure and enhances the ability to lead (Rogelberg et al., 2013). Leaders often use self-talk in combination with other psychological skills, such as planning, rationalization, and visualization, to reduce stress and improve their behavior (Olusoga et al., 2010). High-ranking military officers have used positive self-talk to overcome acute stress from traumatic events (Sahoo & Devashish, 2019). Self-talk has been hypothesized to improve leaders’ effectiveness (Neck et al., 1999) and is essential for performance and coping with stressors in the military context (Meyer, 2018). In sport, positive, motivational, and instructional self-talk can enhance performance (Van Raalte et al., 2016) and help defeat anxiety (Hanton & Jones, 1999). However, excessive instructional self-talk can impair performance (Hardy, 2006) due to “overthinking and cognitive overload” (Van Raalte et al., 2017, pp. 142–143). Moreover, research suggests that positive self-talk (Wood et al., 2009) only slightly increases good mood among those with good self-esteem while negatively affecting those with low self-esteem. As mixed results exist, positive motivational and instructional self-talk generally has encouraging effects on performance and mental health. However, little is known about high-level leaders’ use of self-talk and how it may affect the impact of demanding conditions on their leader performance and mental health.

Another central psychological skill is imagery, which is supposed to enhance leader performance. Research in the military shows that imagery can be effective among recruits exposed to real-life military training in which “performance under pressure holds real consequences for success and failure” (Fitzwater et al., 2018, p. 105). Moreover, leaders in sport use imagery, for example, to control their emotions by thinking through how to reach their athletes (Thelwell et al., 2008).

Thelwell et al. (2008) also found that these elite coaches, to a limited extent, used goalsetting for their performance, emotional control, and focus. Goalsetting within the framework of psychological skills training in the military (Taylor et al., 2011) and sport (Williamson et al., 2022) appears to contribute to better performance. Using goalsetting may thus be related to better leader performance.

Still, another vital psychological skill is mindfulness, which has been shown to positively affect performance among leaders (King & Haar, 2017), military personnel (Jha et al., 2017), and elite athletes (Josefsson et al., 2019). Moreover, mindfulness improves vitality and significantly reduces psychological distress (Canby et al., 2015). Such a decline in distress reflects mindfulness as a practical psychological skill to regulate emotions (Roemer et al., 2015). Finally, research on emotional regulation shows that closely related to mindfulness is cognitive reappraisal (Troy et al., 2013), which can improve well-being (Gross & John, 2003; Larsson et al., 2024) and leader performance (Torrence & Connelly, 2019). Thus, mindfulness and cognitive reappraisal can potentially constitute a unified psychological skill for emotional regulation that is positively related to enhanced mental health and leader performance.

Research questions about Swedish high-ranking military officers and sport executives’ coping strategies, especially psychological skills to maintain their mental health and leader performance under demanding conditions, have been raised because of their shared high-stress and high-stakes leader roles. However, cross-contextual research and repeated measures design are limited in this area. Therefore, based on Adler and Castro’s (2013) model focusing on personal resources, the current cross-contextual repeated measures study aimed to (1) investigate how experienced demanding conditions were related to self-rated leader performance level and mental health indicators among high-level military and sport leaders, and (2) whether the application of psychological skills by these leaders moderated these relationships.

The following predictions were made: (1) higher levels of experienced demanding conditions will be associated with lower levels of leader performance and mental health, and vice versa; (2) higher levels of self-talk, imagery, goalsetting, and emotional regulation represented by mindfulness and cognitive reappraisal will be associated with higher levels of leader performance, and vice versa; and (3) higher levels of self-talk, emotional regulation represented by mindfulness, and cognitive reappraisal, will be associated with higher levels of mental health, and vice versa.

## Method

### Responders, procedure, and ethics

The current study was approved by the Swedish Ethical Review Authority (Dnr: 2022–03789–01). Based on purposive sampling, 53 responders were recruited, of whom one dropped out. The sample of responders who then participated in the study consisted of 52 responders: 26 high-ranking Swedish military officers (Lieutenant Colonel or Commander (CDR)), 13 women and 13 men, and 26 executives in Swedish elite team sport organizations, 13 women and 13 men. The selection criteria were: (1) Lieutenant Colonel/Commander (CDR)/Regimental Sergeant Major/Chief Warrant Officer/Wing Warrant Officer who holds a senior leading position in an organizational unit (interpreted as part of an organization that also includes sections, departments, or projects) in the Swedish Armed Forces; (2) Chairman, Club Director, General Manager/Sport Manager/Head of Operations at a senior position in an association/organization at the top elite level in Swedish team sports organizations; (3) No less than 30% of the responders in each context, military or sport, should be represented by women. The responders were classified into one of five age categories: zero between 20 and 29 years, seven between 30 and 39 years, twenty between 40 and 49 years, twenty-four between 50 and 59 years, and one from 60 years and older. Twenty-five officers had undergone Lieutenant Colonels/Commanders (CDR) higher education, equivalent to MA or MSc academic qualifications. One officer had a different but corresponding academic education. Among the sport executives, a mix of education in sports, business/finance, management, and leadership emerged as most relevant to their executive roles. Twelve sport executives listed academic education, while two reported professional sports manager education. Moreover, five sport executives reported professional management and business education, and three reported elite sports coaching education. One sport executive reported sports folk high school, and three reported high school education in leadership or economy. All military officers and sport executives had extensive leadership experience, predominantly in their present context.

### Design and data collection

#### Demanding conditions

By way of introduction to the questionnaire and its measures, the responders gave a free text answer, describing the main and most demanding leadership challenge they had to maneuver over the past week. To illustrate some of the challenges, a couple of examples are provided below.

My civilian employee base secretary of staff and my base chief of staff collided at the workplace. This partly meant a mediating role for me but also an analysis of what the actual problem was. (Female military leader)

The most challenging issue has been that our executive team currently does not have the trust in each other that is needed to perform at a high level. We value issues differently, and there are challenges that must be solved for us to get where we want to go. My background comes from elite sports, and most others, in my opinion, do not understand the incentives to succeed at the elite level. They have many good thoughts, but more are needed to reach all the way. (Male sport leader)

After the free text response about the main and most demanding leadership challenge in the past week, each responder indicated on a single item how demanding this main leadership challenge was on a 5-point Likert response scale. The scale ranged from 1 (*not at all demanding*) to 5 (*extremely demanding*). Based on the recommendations of Matthews et al. (2022), the first author developed this single-item solution for the current study using response categories that were deemed to be closest to the item construct definition.

#### Leader performance

The leader performance self-rating was conducted after measuring the level of the most demanding leadership challenge. Each responder looked back at the past week and indicated on a single item how well they felt they had performed concerning the most demanding leadership challenge in the last week on a 5-point Likert response scale ranging from 1 (*significantly below my normal performance*) to 5 (*significantly above my normal performance*). This single-item self-rating was explicitly developed for this study by the first author in dialogue with high-ranking military officers who compared performance with what they labeled normal performance. In line with Rossiter (2002), the single-item solution assumed that the responders understood in which context and against which concrete object (“the” most demanding leadership challenge, the last week) they assessed their performance.

#### Mental health

##### Vitality

Each responder reviewed the past week and completed a 6-item modified Swedish version of Ryan and Frederick’s (1997) 7-point subjective vitality scale. The original reversed item 2, “I do not feel very energetic, was excluded. A sample item: “I feel alive and vital.” A 7-point Likert response scale was used, ranging from 1 (*not at all true*) to 7 (*very true*). McDonald’s ω on vitality: T0: 0.900; T1: 0.915; T2: 0.938; and T3: 0.947.

##### Stress symptoms

Each responder reviewed the past week and filled in a 15-item scale measuring physiological (6 items), emotional (6 items), and cognitive stress symptoms (3 items) adopted from the Swedish version of Setterlind and Larsson’s (1995) Stress Profile instrument. Example items: “Headache or migraine,” “Frustration/irritation or anger” (added item), “Difficulty thinking clearly.” A 5-point Likert response scale was used, ranging from 1 (*never*) to 5 (*very often*). McDonald’s ω: Physical stress symptoms, T0: 0.783; T1: 0.810; T2: 0.768; and T3: 0.798; Emotional stress symptoms, T0: 0.853; T1: 0.868; T2: 0.861; and T3: 0.826; Cognitive stress symptoms, T0: 0.838; T1: 0.793; T2: 0.876; and T3: 0.809.

#### Psychological skills

Each responder reviewed the past week and completed eighteen items altogether, measuring the four psychological skills of self-talk, imagery, goal-setting-mental-preparation, and emotional regulation. Each responder reviewed the past week and completed eighteen items. The items were extracted from different psychological skills instruments (see below). A 5-point Likert response scale was used for all items, ranging from 1 (*never*) to 5 (*always*).

##### Self-talk

For measuring self-talk, eight items were extracted and modified from the Zervas et al. (2007) self-talk questionnaire (ST-Q), measuring cognitive-instructional self-talk, for example, “I am having an inner conversation with myself to be able to concentrate more fully on my leader performance,” and motivational self-talk, for example, “I am having an inner conversation with myself to encourage myself.” McDonald’s ω: T0: 0.863; T1: 0.887; T2: 0.917; and T3: 0.918.

##### Imagery

Two items from the Test of Performance Strategies-64 (TOPS) (Thomas et al., 1999) were extracted to measure imagery; for example, “I visualize my leader performance exactly as I want it to be.” McDonald’s ω: T0: 0.823; T1: 0.781; T2: 0.784; and T3: 0.795.

##### Goalsetting-mental preparation

Three items were taken from a Swedish version of the Athletic Coping Skills Questionnaire-28 (ASCI) (Smith et al., 1995) to measure goalsetting-mental preparation. Example item: “Daily or weekly, I analyze the situation and set specific goals for myself that guide what I do as a leader.” McDonald’s ω: T0: 0.800; T1: 0.816; T2: 0.803; and T3: 0.847.

##### Emotional regulation

To assess emotional regulation, two items were extracted and modified from a Swedish version of the Five Facet Mindfulness Questionnaire (FFMQ) (Baer et al., 2006). Example item: “When I have emotionally upset thoughts or images of a demanding leadership challenge, I take a step back and am aware of the thought or image without being overwhelmed by it.” Also, three items were taken and modified from the Emotion Regulation Questionnaire (ERQ) (Gross & John, 2003); for example, “When I want to feel less negative emotions, I change the way I think about the demanding leadership challenge.” McDonald’s ω: T0: 0.712; T1: 0.716; T2: 0.777; and T3: 0.817.

#### Data analysis

The data were analyzed in Mplus (8.4) within the Bayesian two-level analysis framework, where the repeated data from each responder (level 1) were nested in responders (level 2). This analysis specified at level 1 the relationships between the independent variable, “the most demanding leadership challenge in the past week, and the dependent/outcome variables of leader performance and mental health. Separate two-level analyses were conducted for each of the outcomes. All four psychological skills variables, collected at Time 0 (baseline), were included as moderators on level 2. Also, gender and context were included as moderators on the same level (level 2). Compared with the traditional frequentist approach, the Bayesian two-level approach is preferable for small sample sizes due to different statistical assumptions (e.g., Stenling et al., 2015). More specifically, due to the less-restrictive distributional assumptions, the normality assumption does not need to be fulfilled to perform the analyses within the Bayesian approach (Yuan & MacKinnon, 2009). The authors performed two hundred thousand iterations using Markov Chain Monte Carlo simulation procedures with a Gibbs sampler. A potential scale reduction factor of around 1 was considered an indicator of convergence (Kaplan & Depaoli, 2012). Moreover, the authors estimated a 95% credibility interval (CI) for all parameters within the models, followed the recommendations by Zyphur and Oswald (2015), and rejected the null hypothesis if the 95% CI did not include zero. JASP 0.17.2.1 was used to calculate descriptive statistics.

## Results

One of the responders was excluded because there was no self-reporting at any measurement point. The data for analysis were then based on the 52 responders described at the beginning of the method section. The descriptive statistics in Table 1 show means and standard deviations among the ten study variables over time. As can be seen, the level of the first three variables – demanding conditions, leader performance, and vitality is relatively moderate over time. The mental health indicators of stress symptoms (study variables four, five, and six) are generally low over time, where the emotional stress level stands out as slightly elevated compared to the cognitive and physical stress levels, of which the latter is the lowest. The intraclass correlations for all six variables presented above ranged between 0.21 (leader performance) and 0.79 (physical stress levels), indicating variances on both within-person as well as between-person levels. Independent Samples T-tests revealed no statistically significant differences in these six variables – demanding conditions and leader performance, nor in vitality, physical, emotional, or cognitive stress symptoms, between high-level military and sport leaders at Time 0 (baseline). At baseline, there were also no significant differences in these six variables between women and men. Regarding the four variables of psychological skills, these are moderately applied over time, of which emotional regulation appears to be the most used and imagery the least.

**Table 1.** Means and standard deviations for study variables over time.

	T0		T1		T2		T3	
*Variables*	*M*	*SD*	*M*	*SD*	*M*	*SD*	*M*	*SD*
***Sub variables***								
***Demanding conditions***	*3,35*	*0,81*	*3,46*	*0,96*	*3,06*	*1,01*	*3,16*	*0.93*
***Leader performance***	*3,11*	*0,47*	*3,04*	*0,62*	*3,04*	*0,60*	*3,06*	*0,55*
***Mental health*** *Vitality*	*4,21*	*1,08*	*3,97*	*1,01*	*3,94*	*1,18*	*3,89*	*1,18*
*Physiological stress symptoms*	*1,73*	*0,65*	*1,66*	*0,63*	*1,63*	*0,64*	*1,61*	*0,62*
*Emotional stress symptoms*	*2,37*	*0,72*	*2,26*	*0,75*	*2,23*	*0,73*	*2,18*	*0,67*
*Cognitive stress symptoms*	*2,08*	*0,67*	*2,08*	*0,69*	*2,07*	*0,78*	*2,05*	*0,66*
***Psychological skills*** *Self-talk*	*2,75*	*0,71*	*2,73*	*0,68*	*2,73*	*0,74*	*2,65*	*0,72*
*Imagery*	*2,37*	*0,90*	*2,47*	*0,87*	*2,56*	*0,92*	*2,50*	*0,88*
*Goalsetting/mental preparation*	*2,78*	*0,85*	*2,68*	*0,91*	*2,75*	*0,84*	*2,71*	*0,81*
*Emotional regulation*	*3,00*	*0,56*	*2,87*	*0,49*	*2,84*	*0,58*	*2,83*	*0,59*

The Bayesian two-level analysis of the data showed the following results about the relationships between the independent variable, “the most demanding leadership challenge in the past week,” and the dependent/outcome variables of leader performance and mental health, and the psychological skills variables as moderators:

### Leader performance

The results showed, on within-person level, no credible effect of the perceived level of demanding conditions on the perceived level of leader performance (*β* = −.02, 95% CI [−.11, .07]). On between-person level, the potential moderators could explain 14.6% of the variance in leader performance. However, none of the moderators had a credible effect on the relationship between the perceived level of demanding conditions and the perceived level of leader performance (*βs* ranging between .04 to .19).

### Mental health

The following results show the relationship between the perceived level of demanding conditions and the perceived level of vitality, physiological, emotional, and cognitive stress symptoms, and psychological skills associated with these relationships.

#### Vitality

On within-person level, the result showed a credible negative relationship between the perceived level of demanding conditions and vitality (*β* = −.15, 95% CI [−.24, −.05]). More specifically, the perceived level of demanding conditions could explain 5.7% of the variance in vitality. The included moderators on between person level could explain 27.6% of the variance in vitality. Self-talk had a credible negative association on the relationship between the perceived level of demanding conditions and vitality (*β* = −.25, 95% CI [−.44, −.03]), while emotional regulation had the opposite relationship (*β* = .32, 95% CI [.09, .50]). None of the other variables moderated the relationship (*βs* ranging between .02 to .14).

#### Physiological stress symptoms

The results showed that the perceived level of demanding conditions had, on within-person level, a positive and credible association with physiological stress symptoms (*β* = .15, 95% CI [.05, .24], R^2^ = .08). There was also a negative credible association between demanding conditions and vitality on between-person level. On the between-person level, the included moderators could explain 15.6% of the variance in physiological stress symptoms. Only self-talk had a credible and positive effect on the association between the perceived level of demanding conditions and physiological stress (*β =* .21, 95% CI [.01, .39]). The results showed no other credible moderators for this specific relationship (*βs* ranging between .01 to .14).

#### Emotional stress symptoms

The perceived level of demanding conditions had, on within-person level, a credible positive relationship with emotional stress symptoms (*β* = .31, 95% CI [.23, .39]) and could explain 14.8% of the variance. On between-person level, only self-talk had a positive, and credible, effect on the relationship between the perceived level of demanding condition and emotional stress symptoms (*β* = .29, 95% CI [.08, .48]). The results showed no other credible moderators for this specific relationship (*βs* ranging between .002 to .09).

#### Cognitive stress symptoms

The perceived level of demanding conditions had, on within-person level, a credible positive relationship with cognitive stress symptoms (*β* = .24, 95% CI [.15, .32], R^2^ = .10). There was also a credible association between demanding conditions and vitality on between-person level. On between-person level, only self-talk had a positive and credible effect on the relationship between perceived level of demanding conditions and cognitive stress symptoms (*β* = .29, 95% CI [.08, .46]). The results showed no other credible moderators for this specific relationship (*βs* ranging between .03 to .18).

## Discussion

This cross-contextual repeated measures study examined the impact of demanding conditions on the leader performance and mental health of Swedish high-level military and sport leaders and whether their psychological skills moderated this impact. Viewed through the lens of Adler and Castro’s (2013) occupational health model of military mental health, key findings showed that, contrary to *prediction 1*, elevated levels of demanding conditions did not affect self-rated leader performance. However, consistent with *prediction 1*, they had a negative effect on mental health. Moreover, unexpectedly, and in contrast to *prediction 2*, none of the psychological skills played any role in the relationship between demanding conditions and leader performance. Additionally, contrary to *prediction 3*, it was found that the psychological skill of self-talk surprisingly strengthened the negative effect of a heightened level of demanding conditions on mental health. All three types of stress symptoms got worse, and vitality was impaired. However, in line with *prediction 3*, mindfulness and cognitive reappraisal, as a unified psychological skill for emotional regulation, positively affected the mental health indicators of vitality compared to self-talk. Two overall conclusions can be drawn: (1) High-level military and sport leaders continue to perform regardless of demanding leadership challenges and impaired mental health; (2) High-level military and sport leaders’ use of mindfulness and cognitive reappraisal as a unified psychological skill to regulate their emotions results in significant positive effects on their vitality when the leadership challenges become more demanding. On the other hand, the application of self-talk has the reverse effects on all mental health indicators.

With *prediction 1* as the point of departure for the interpretation of the findings, although previous research (e.g., Frey, 2007) found that the stresses of demanding conditions decline self-rated leader performance, the current study’s simultaneous focus on high-level military and sport leaders showed neither a decline nor an improvement in leader performance. One potential explanation for these findings could be that the high-level leaders in the current study are, according to Fiedler (2001), so well experienced that they recognize the leadership challenge and know how to perform even if it becomes more demanding. Therefore, their performance seems stable over time and is not significantly affected by the most demanding leadership challenges they experience during a typical month. It is possible that the leader’s performance would be affected under more challenging, unfamiliar, and exceptionally demanding conditions. However, a few leaders experienced the “typical month” as somewhat extreme. Nevertheless, the average level of demanding conditions among the responders ranged from 3.06 to 3.46 (see Table 1), which we interpreted as not more than moderately demanding.

Even if demanding conditions did not affect leader performance, they harmed the responders’ mental health in terms of diminished vitality and increased stress symptoms. This is consistent with previous research showing that high-level military and sport leaders’ most demanding conditions are primarily manifested in severe stress reactions, although these conditions can be experienced as exciting (Bencker et al., 2022). It seems that the quantitative results in the current study and the qualitative results in the previous study point in the same direction. That is, when high-level leaders perceive their environment consists of demanding conditions with a series of inherent leadership challenges, they can be severe enough to be manifested in greater intrapersonal stress levels and mental health issues like reduced vitality. Similarly, professional first responders (Larsson et al., 2016), military veterans (Larsson et al., 2021), and sport leaders (Potts et al., 2023; Richman, 1992) have reported decreased well-being as daily hassles and pressures increase. It appears that senior sport executives and high-ranking military officers are susceptible to experiencing stress at some point, even if they adapt positively to challenges (Baldock et al., 2022), are hardy (Kokun et al., 2023), and that other senior leaders alike, report higher levels of flourishing and life satisfaction in the face of challenges (Wallis et al., 2021). The fact that the responders’ mental health declines when the pressures of their most demanding leadership challenges become too great may imply that there may be other types of pressures and daily hassles that need to be limited, when possible, to give the leaders a better chance to stay vital, feel good, and have more energy and time to maneuver their leadership challenges.

Concerning *prediction 2*, the findings showed that the use of psychological skills like imagery and setting goals as personal resources to manage the effect of the responders’ appraised leadership challenges on their mental health and performance, as described in the occupational health model for military mental health (Adler & Castro, 2013), did not moderate the relationship between demanding conditions and leader performance.

These findings were unexpected as they contradict previous research on self-talk (Rogelberg et al., 2013), visualization (Olusoga et al., 2010), goalsetting (Thelwell et al., 2008), mindfulness (King & Haar, 2017), and cognitive reappraisal (Torrence & Connelly, 2019), all of which were reported to benefit leader performance. On the other hand, previous research shows, for example, that excessive self-talk can impair performance (Hardy, 2006). It is possible that the responders are performing at a normal level that is already high and that it is, therefore, difficult for them to perform even better by using psychological skills as a personal resource. This may indicate a measurement problem in terms of a ceiling effect – a limited performance improvement beyond normal performance for already professional, high-performing leaders at this high organizational leader level (Larsson et al., 2018). However, it is also possible that the demanding conditions, as previously mentioned, were not exceptionally demanding, that they were not of the kind that Adler and Castro (2013) refer to as high-risk/traumatic work situations, that may have challenged the responders’ leader performance. Therefore, it is possible that the responders were able to cope with their challenges without affecting their performance. Perhaps the responders were frustrated that the challenges took their time away from other important matters, created leader role overload or irritation, or were unexpected. However, this does not necessarily mean that the demanding leadership challenges, during the limited period of one month, required high amounts of psychological skills i.e. cognitive resources and strategies, or new leadership capabilities as a true test of responders’ ability to perform successfully as leaders. In line with Sherman et al. (2012) and Kokun et al. (2023), this may mean that the responders were in control and too experienced for their performance to deteriorate, and not in need of an increased use of cognitive strategies, relative to the degree of demanding conditions they experienced.

Due to *prediction 3*, an unforeseen finding was that instructional and motivational self-talk as a moderating psychological skill surprisingly strengthened the negative effect of a heightened level of demanding conditions on mental health. This challenges previous research showing self-talk as a valuable psychological skill for coping with stressors in the military (e.g., Meyer, 2018), and defeating anxiety (Hanton & Jones, 1999) in sport. Nevertheless, there is empirical evidence showing that positive self-talk can worsen mood (Wood et al., 2009), which is reminiscent of the findings of exacerbated emotional stress symptoms in the present study. A possible explanation for self-talk’s negative moderating effect is that too much of an instructive and motivating inner dialogue under demanding leadership challenges leads to unhealthy “overthinking and cognitive overload” (Van Raalte et al., 2017, pp. 142–143). This may reflect the increased cognitive stress symptoms, such as difficulty thinking clearly, which are assumed to be closely connected with the exacerbated emotional and physiological stress symptoms and decreased vitality.

In contrast to the unexpected findings on self-talk and in line with *prediction 3*, mindfulness and cognitive reappraisal as a unified psychological skill for emotional regulation had a positive effect on the mental health indicators of vitality compared to self-talk. This resonates with previous research indicating that mindfulness improves vitality (Canby et al., 2015) and, together with cognitive reappraisal, enhances well-being (Gross & John, 2003). The findings coincide with previous research showing that mindfulness is a practical psychological skill for emotion regulation (Roemer et al., 2015) and, in close association with cognitive reappraisal (Troy et al., 2013), forms an effective unison psychological skill for increased vitality among high-level military and sport leaders when performing under demanding conditions.

As instructional and motivational self-talk strengthened the negative effect of a heightened level of demanding conditions on mental health, these findings suggest that applying increased levels of self-talk does not aid high-level military and sport leaders in feeling good. However, according to the findings, what does aid those leaders in feeling revitalized is their use of mindfulness and cognitive reappraisal as a unified psychological skill for emotional regulation. These divergent findings regarding the moderating role of psychological skills suggest emphasizing their nuanced application and avoiding a one-sided belief in their positive effects on high-level leaders’ performance and mental health under demanding conditions. A de-emphasizing of self-talk and an up-emphasizing of the development and application of mindfulness and cognitive reappraisal are suggested. This may contribute to high-level leaders’ more robust set of psychological skills, as the latter has been highlighted in the military as beneficial for mental health (McGraw et al., 2012) and as important for leaders in sport to better deal with stress (Norris et al., 2017). However, this does not mean that other psychological skills, such as imagery and goalsetting, should be neglected because research shows they can be suitable for some leaders (e.g., Rogelberg et al., 2013). Intervention among high-level leaders is suggested to adopt an increased focus on vitality and related psychological skills to make leaders, in addition to performing well, feel better under demanding leadership challenges.

The strengths of the current study were the longitudinal research design, the low drop-out rate during the four weeks of repeated measures, and the symmetrical composition of responders regarding military versus sport and females versus males, which increased the context- and gender-crossing relevance of the findings. A potential methodological weakness was the relatively small sample (*n* = 52). On the other hand, this was compensated by the repeated measures design and the Bayesian two-level approach, which are preferable for small sample sizes due to different statistical assumptions (e.g., Stenling et al., 2015). Moreover, the current measure of leader performance needs to be addressed. There are criticisms against self-reporting of performance, of which probably the best known is self-overestimation due to the need for social desirability (e.g., Moorman & Podsakoff, 1992). At the same time, confidentiality in research lowers the tendency toward self-enhancement in self-reports (Mabe & West, 1982). Moreover, people are assumed to be capable enough to evaluate their performance accurately through their experience and understanding of their work roles (Van der Heijden & Nijhof, 2004). In line with Rossiter (2002), the use of a single-item solution for self-reported leader performance assumed that the responders know in what context and against what concrete object (“the” most difficult leadership challenge in the last week) they judged their level of performance as a leader. Hence, the current study has confidence in the single-item solution of self-reported performance. Regarding the measure of psychological skills, there was a risk that reliability was compromised due to the items being modified with relevance to the population and context. Nevertheless, McDonald’s score for all psychological skills was over 0.7.

More cross-contextual leadership research, suggestively applied research, is needed to better understand the links between high-level military and sport leaders’ psychological skills, leader performance, and mental health under demanding conditions. Perhaps mixed-methods research (MMR) with qualitative free-text answers would be suitable for accessing nuances and dynamics in psychological skills (Sparkes, 2015), mental health, and leader performance (Stentz et al., 2012) to learn more about these variables among high-level leaders. Moreover, as the measures of psychological skills in this study were based on item extraction from previously validated questionnaires (Baer et al., 2006; Smith et al., 1995; Thomas et al., 1999; Zervas et al., 2007) and modification of these items, further psychometric development of these measures is suggested for improved adaptation to high-level military and sport leaders.

Considering future research due to the findings in the current study, the effect of demanding conditions on leaders’ mental health may not depend solely on the severity of demanding conditions and the application of psychological skills such as emotional regulation. Based on Adler and Castro’s (2013) model of military mental health, research on the relationship between demanding conditions, mental health, and leader performance can therefore also look at other factors like occupational resources such as training and intervention, the leadership of the leaders’ superiors, as well as the personal resources of physical training, social support, and other mental concepts such as hardiness. Research on both psychological skills and those other factors and concepts can promote a more holistic knowledge of high-level leaders’ resources, capabilities, and strategies for maintaining mental health and performance under demanding conditions.

Moreover, future research can include a more heterogeneous sample of military officers and sports leaders, also focusing on their backgrounds, as this may give different results in accordance with Adler and Castro’s (2013) model, which points out that a leader’s experience and rank are claimed to be a basis for better mental health. Finally, future research should also address potential differences related to Adler and Castro’s (2013) differentiation between traditional occupational, and high-risk/traumatic work situations. In the present study, all reported demanding leadership challenges could be described as traditional occupational.

The current study discovered that demanding conditions do not negatively impact the leader performance of high-level military and sport leaders. However, they can negatively impact the leaders’ mental health, resulting in diminished vitality and increased stress symptoms. Unexpectedly, the leaders’ application of instructional and motivational self-talk exacerbates these stress symptoms and further decreases vitality. In contrast, the leaders’ use of mindfulness and cognitive reappraisal as a unified psychological skill to manage emotions favors vitality as the leadership challenges become more demanding. The study’s sample symmetry highlights the findings’ context- and gender-crossing strengths. Further cross-contextual, suggestively applied research on high-level military and sport leaders is needed to better understand the connections between psychological skills, leader performance, and mental health under demanding conditions.

## Data Availability

Data are available from the first author upon request.
